# Oral Bisphosphonate Exposure and the Risk of Upper Gastrointestinal Cancers

**DOI:** 10.1371/journal.pone.0140180

**Published:** 2015-10-07

**Authors:** Emily Vogtmann, Douglas A. Corley, Lucy M. Almers, Chris R. Cardwell, Liam J. Murray, Christian C. Abnet

**Affiliations:** 1 Nutritional Epidemiology Branch, Division of Cancer Epidemiology and Genetics, National Cancer Institute, National Institutes of Health, Bethesda, Maryland, United States of America; 2 Cancer Prevention Fellowship Program, Division of Cancer Prevention, National Cancer Institute, National Institutes of Health, Bethesda, Maryland, United States of America; 3 Division of Research, Kaiser Permanente, Oakland, California, United States of America; 4 Centre for Public Health, Queen’s University Belfast, Belfast, United Kingdom; College of Medicine, National Taiwan University, TAIWAN

## Abstract

The association between oral bisphosphonate use and upper gastrointestinal cancer has been controversial. Therefore, we examined the association with esophageal and gastric cancer within the Kaiser Permanente, Northern California population. A total of 1,011 cases of esophageal (squamous cell carcinoma and adenocarcinoma) and 1,923 cases of gastric adenocarcinoma (cardia, non-cardia and other) diagnosed between 1997 and 2011 from the Kaiser Permanente, Northern California cancer registry were matched to 49,886 and 93,747 controls, respectively. Oral bisphosphonate prescription fills at least one year prior to the index date were extracted. Conditional logistic regression was used to calculate odds ratios (OR) and 95% confidence intervals (95% CI) for the associations between prospectively evaluated oral bisphosphonate use with incident esophageal and gastric cancer diagnoses with adjustment for potential confounders. After adjustment for potential confounders, no significant associations were found for esophageal squamous cell carcinoma (OR 0.88; 95% CI: 0.51, 1.52), esophageal adenocarcinoma (OR 0.68; 95% CI: 0.37, 1.24), or gastric non-cardia adenocarcinoma (OR 0.83, 95% CI: 0.59, 1.18), but we observed an adverse association with gastric cardia adenocarcinoma (OR 1.64; 95% CI: 1.07, 2.50). In conclusion, we observed no association between oral bisphosphonate use and esophageal cancer risk within a large community-based population. A significant association was detected with gastric cardia and other adenocarcinoma risk, although this needs to be replicated.

## Introduction

Osteoporosis is an important global issue with the growing aging population. In the United States, an estimated 9 million adults have osteoporosis and at least 48 million adults have an increased risk of osteoporosis related to low bone mass [1]. Bisphosphonates, a class of drugs which decrease osteoclast-mediated bone resorption, are often prescribed to prevent and treat osteoporosis [2]. When not taken as instructed (i.e., lying down within 30 minutes of taking the medication), oral bisphosphonates can cause injury to the esophageal mucosa resulting in complications such as bleeding or ulcerations [3, 4]. In 2009, a US Food and Drug Administration case report suggested an association between bisphosphonates and risk of esophageal cancer, potentially related to bisphosphonate induced esophageal mucosal injury [5].

Epidemiological studies of the association between bisphosphonates and upper gastrointestinal cancer have been inconsistent and these studies have generally not considered histological or site-specific information for esophageal or gastric cancer [6–16]. Therefore, using a case-control design, we assessed the potential association between oral bisphosphonate use and upper gastrointestinal cancer within a large population which had not been previously utilized to assess this association. This dataset additionally included important information on cancer histology in order to address histological and site specific associations with bisphosphonates.

## Materials and Methods

### Source population

Cases and controls were selected from adult members (≥ 18 years old) of the Kaiser Permanente, Northern California health system from 1997 to 2011. Kaiser Permanente is California’s largest non-profit health plan and currently has approximately 3.3 million members in northern California who are generally representative of the general population in that region [17]. This study was approved by the Kaiser Permanente, Northern California Institutional Review Board which waived the requirement for written informed consent. All patient data was anonymized and de-identified and this study was considered not human subjects research by the National Cancer Institute.

### Case identification

We selected cases of invasive esophageal (ICD–10: C15) and gastric (ICD–10: C16) cancer from the Kaiser Permanente, Northern California cancer registry and assigned the date of the diagnosis as the index date. Cases had to be at least 18 years old and have at least 2 years of membership in Kaiser Permanente, Northern California prior to diagnosis. We excluded cases with a history of cancer prior to the index cancer (as indicated in the cancer registry or ICD–9 V codes) or a history of Paget’s disease (ICD–9: 731.0). Using these criteria, we identified 1,011 cases of esophageal and 1,923 cases of gastric cancer. Since the etiology of the two main histologic types of esophageal cancer differ, cases were further classified as esophageal squamous cell carcinoma (ICD-O–3 histology: 8070, 8071, 8094) or adenocarcinoma (ICD-O–3 histology: 8140, 8144, 8210, 8481, 8490). Similarly, gastric cancer cases were divided by site which included gastric cardia (ICD-O–3 site: C16.0), non-cardia (ICD-O–3 site: C16.1, C16.2, C16.3, C16.4, C16.5, C16.6, C16.7), and other adenocarcinoma including overlapping (ICD-O–3 site: C16.8) and unspecified (ICD-O–3 site: C16.9) sub-sites. All gastric cancers were adenocarcinoma.

### Control selection

Up to 50 controls from the Kaiser Permanente, Northern California database were matched without replacement to each case on gender, age at time of index date (+/- 2 years), duration of membership prior to index date (+/- 1 year), race, and region of residence. To be eligible, the control had to be at least 18 years of age and a member of Kaiser Permanente, Northern California at the index date for the matched case and membership for at least two years prior to the index date for the matched case. A total of 49,886 and 93,747 controls were identified for the cases of esophageal and gastric cancer, respectively.

### Oral bisphosphonate exposure assessment

Data on oral bisphosphonate prescription fills were collected prior to the index date for both cases and controls from the Kaiser Permanente, Northern California database. We categorized exposure to oral bisphosphonates as ever (at least one prescription fill at least one year prior to the index date) versus never. Among participants exposed to bisphosphonates, we calculated defined daily dose of bisphosphonate exposure prior to the index date and categorized this as less than 12 months of exposures and 12 or more months of exposure [18].

### Other covariates of interest

Data on other covariates prior to the index date were collected from the Kaiser Permanente, Northern California database including demographic variables (age, sex, and race/ethnicity), history of smoking and alcohol consumption, body mass index (BMI) and the Charlson Comorbidity Index (CCI) [19]. Diagnoses for other comorbidities including Barrett’s esophagus, dyspepsia, esophagitis, gastroesophageal reflux disease (GERD), and osteoporosis were included if identified at least one year prior to the index date. We determined which participants had a prescription drug fill at least one year prior to the index date for proton pump inhibitors (PPIs), H2-receptor antagonists, hormone replacement therapy (HRT), and non-steroidal anti-inflammatory drugs (NSAIDs). A history of receiving an upper or lower endoscopy at least one year prior to the index date was also ascertained. All participants had complete data for all covariates with the exception of BMI (52% missing), so the BMI data were only used in sensitivity analyses.

### Statistical analysis

#### Primary analysis

We described the esophageal cancer cases by histologic type, gastric cancer cases by site, and the combined controls. We calculated odds ratios (ORs) and 95% confidence intervals (95% CIs) for the associations between oral bisphosphonate use and esophageal and gastric cancer using conditional logistic regression with adjustment for age, smoking status, alcohol use, CCI, use of PPIs, NSAIDs, or H2-receptor antagonists, and history of GERD, osteoporosis, or upper endoscopy. We tested for an interaction between bisphosphonate use and sex on the risk of esophageal and gastric cancer by breaking the matched pairs and including interaction terms in an unconditional logistic regression model.

#### Secondary analyses

As a sensitivity analysis, we created conditional logistic regression models within the population with valid BMI data and tested the effect of the inclusion of BMI in model adjustment. We additionally created a model within participants who had no indication of GERD (no diagnosis of GERD or a prescription for H2-receptor antagonists or PPIs) and a model within participants who had not received an upper endoscopy at least one year prior to the index date. All analyses were conducted using SAS 9.3 and a two-sided p-value of 0.05 was considered statistically significant.

## Results

Demographic characteristics of the esophageal cancer cases, gastric cancer cases, and controls are presented in Table 1. As expected, esophageal squamous cell carcinoma cases were more likely to have smoked tobacco (67.0%) and to have consumed alcohol (51.9%) than the esophageal cancer controls (39.3% and 23.2%, respectively).

**Table 1:** Descriptive characteristics of the esophageal and gastric cancer cases and controls from Kaiser Permanente, Northern California, 1997–2011.

	Esophageal cancer			Gastric cancer			
	SCC	Adenocarcinoma	Control	Cardia	Non-Cardia	Other	Control
	%/Mean (SD)	%/Mean (SD)	%/Mean (SD)	%/Mean (SD)	%/Mean (SD)	%/Mean (SD)	%/Mean (SD)
Number of participants	385	626	49,886	626	915	382	93,747
Ever oral bisphosphonates	4.2%	1.9%	3.1%	4.5%	4.4%	6.8%	4.2%
Defined Daily Dose							
Never	95.8%	98.1%	96.9%	95.5%	95.6%	93.2%	95.8%
< 12 months	2.3%	1.0%	1.1%	1.6%	1.3%	2.6%	1.5%
>= 12 months	1.8%	1.0%	2.0%	2.9%	3.1%	4.2%	2.7%
Age at index	68.7 (10.8)	66.5 (11.4)	67.2 (11.1)	66.0 (11.7)	68.9(13.1)	66.5 (13.6)	67.2 (12.7)
Female	36.6%	12.9%	22.1%	20.8%	42.6%	41.4%	35.5%
Race/ethnicity							
Non-Hispanic white	57.7%	80.0%	72.3%	72.8%	34.4%	35.6%	48.2%
Non-Hispanic black	14.8%	1.4%	6.3%	3.5%	13.6%	13.6%	9.9%
Hispanic	9.4%	10.7%	10.1%	12.0%	27.2%	27.7%	22.2%
Other	17.9%	7.7%	11.0%	11.5%	24.7%	22.8%	19.5%
Missing	0.3%	0.2%	0.2%	0.2%	0.1%	0.3%	0.2%
Smoked tobacco (% yes)	67.0%	61.5%	39.3%	51.6%	44.9%	38.2%	35.0%
Consumed alcohol (% yes)	51.9%	35.5%	23.2%	28.0%	24.8%	22.3%	19.8%
Body mass index							
Missing	55.6%	46.3%	49.5%	54.5%	52.3%	53.9%	53.8%
Underweight	1.0%	0.0%	0.1%	0.0%	0.2%	0.0%	0.1%
Normal weight	23.4%	12.3%	15.8%	11.5%	16.8%	17.8%	16.1%
Overweight	11.2%	15.8%	17.3%	13.7%	15.2%	14.1%	15.3%
Obese	8.8%	25.6%	17.4%	20.3%	15.4%	14.1%	14.7%
CCI	0.8 (1.9)	0.6 (1.6)	0.2 (0.8)	0.8 (1.9)	1.4 (2.3)	1.3 (2.4)	0.2 (0.8)
History of Barrett's esophagus	0.5%	9.1%	0.5%	2.2%	0.4%	0.3%	0.5%
History of dyspepsia	7.5%	6.9%	7.6%	6.5%	13.4%	12.3%	8.6%
History of esophagitis	3.4%	6.7%	2.3%	2.9%	2.3%	2.1%	2.1%
History of GERD	21.6%	34.7%	22.0%	24.3%	26.3%	20.4%	21.4%
History of osteoporosis	42.3%	49.0%	47.9%	44.1%	45.7%	42.7%	46.8%
Previous lower endoscopy	40.0%	43.8%	45.6%	41.1%	40.0%	35.9%	41.8%
Previous upper endoscopy	11.2%	10.4%	6.4%	5.8%	6.7%	7.6%	6.2%
Ever PPIs	22.3%	28.0%	18.9%	21.4%	24.7%	20.4%	18.5%
Ever H2-RAs	29.9%	39.8%	33.3%	32.3%	43.9%	36.4%	34.6%
Ever HRT	14.8%	7.5%	12.5%	10.1%	19.5%	16.8%	17.1%
Ever NSAIDs	66.0%	67.9%	69.7%	66.9%	67.0%	63.1%	70.3%

Abbreviations: Charlson Comorbidity Index (CCI), gastroesophageal reflux disease (GERD), proton pump inhibitors (PPIs), H2-receptor antagonists (H2-RAs), hormone replacement therapy (HRT), non-steroidal anti-inflammatory drugs (NSAIDs), squamous cell carcinoma (SCC)

Exposure to oral bisphosphonates was similar for esophageal squamous cell carcinoma and adenocarcinoma cases compared to their matched controls (4.2% and 1.9% versus 4.3% and 2.4%, respectively). Gastric cardia and other gastric adenocarcinoma cases tended to have a non-significant increase to oral bisphosphonates (4.5% and 6.8% versus 2.9% and 4.2%, respectively) while the non-cardia gastric adenocarcinoma cases did not have a higher prevalence of exposure to bisphosphonates compared to their matched controls (4.4% versus 5.0%, Table 2).

**Table 2:** Conditional logistic regression for the associations between oral bisphosphonates and esophageal or gastric cancer in Kaiser Permanente, Northern California, 1997–2011.

		Cases	Controls	Unadjusted OR	Adjusted OR
	Bisphosphonate use	N (%)	N (%)	(95% CI)	(95% CI)
**Esophageal cancer**					
Squamous cell carcinoma	Never	369 (95.8%)	18,024 (95.7%)	Ref	Ref
	Ever	16 (4.2%)	808 (4.3%)	0.96 (0.56, 1.65)	0.92 (0.53, 1.60)
	< 12 months	9 (2.3%)	289 (1.5%)	1.52 (0.76, 3.02)	1.27 (0.63, 2.58)
	≥ 12 months	7 (1.8%)	519 (2.8%)	0.65 (0.30, 1.41)	0.68 (0.31, 1.50)
Adenocarcinoma	Never	614 (98.1%)	30,316 (97.6%)	Ref	Ref
	Ever	12 (1.9%)	738 (2.4%)	0.79 (0.43, 1.43)	0.68 (0.37, 1.24)
	< 12 months	6 (1.0%)	278 (0.9%)	1.05 (0.46, 2.40)	0.84 (0.36, 1.92)
	≥ 12 months	6 (1.0%)	460 (1.5%)	0.62 (0.27, 1.44)	0.57 (0.25, 1.32)
**Gastric cancer**					
Cardia	Never	598 (95.5%)	29,951 (97.1%)	Ref	Ref
	Ever	28 (4.5%)	900 (2.9%)	1.61 (1.05, 2.45)	1.67 (1.09, 2.56)
	< 12 months	10 (1.6%)	322 (1.0%)	1.64 (0.85, 3.15)	1.58 (0.81, 3.06)
	≥ 12 months	18 (2.9%)	578 (1.9%)	1.59 (0.95, 2.65)	1.73 (1.03, 2.90)
Non-cardia	Never	875 (95.6%)	42,027 (95.0%)	Ref	Ref
	Ever	40 (4.4%)	2,224 (5.0%)	0.85 (0.60, 1.20)	0.84 (0.59, 1.20)
	< 12 months	12 (1.3%)	796 (1.8%)	0.72 (0.40, 1.29)	0.63 (0.34, 1.15)
	≥ 12 months	28 (3.1%)	1,428 (3.2%)	0.93 (0.62, 1.39)	0.99 (0.65, 1.48)
Other	Never	356 (93.2%)	17,856 (95.8%)	Ref	Ref
	Ever	26 (6.8%)	789 (4.2%)	1.81 (1.16, 2.84)	1.93 (1.22, 3.06)
	< 12 months	10 (2.6%)	285 (1.5%)	1.94 (1.00, 3.77)	1.91 (0.95, 3.82)
	≥ 12 months	16 (4.2%)	504 (2.7%)	1.74 (1.01, 3.01)	1.94 (1.12, 3.38)

Cases and controls were matched on gender, age at time of index date (+/- 2 years), duration of membership prior to index date (+/- 1 year), race, and region of residence. The adjusted conditional logistic regression models additionally adjusted for age, smoking, alcohol use, CCI, use of PPIs, NSAIDs, or H2-Receptor antagonists, history of GERD or osteoporosis, and previous upper endoscopy.

No association was detected between oral bisphosphonate use at least one year prior to the index date for esophageal squamous cell carcinoma (adjusted OR 0.92; 95% CI: 0.53, 1.60) or adenocarcinoma (adjusted OR 0.68; 95% CI: 0.37, 1.24). Similarly, no consistent association was detected for duration of exposure to oral bisphosphonates with esophageal cancer. A positive association was detected between oral bisphosphonate use and gastric cardia (adjusted OR 1.67; 95% CI: 1.09, 2.56) and other gastric adenocarcinoma (adjusted OR 1.93; 95% CI: 1.22, 3.06), while no significant association was observed with non-cardia gastric adenocarcinoma (adjusted OR 0.84; 95% CI: 0.59, 1.20). The associations with gastric cardia and other gastric adenocarcinoma were slightly strengthened for participants who had been exposed to oral bisphosphonates for a year or more (Table 2). No interactions were detected between oral bisphosphonate use and sex on any cancer outcome (p > 0.05; results not shown).

### Secondary analyses

When we restricted the analyses to participants with BMI data, the estimates with and without adjustment for BMI were relatively unchanged (results not shown). Exclusion of participants with a prior GERD diagnosis produced nonsignificant trends for positive associations for gastric cardia (OR 1.95; 95% CI: 0.98, 3.89) and other gastric (OR 1.77; 95% CI: 0.86, 3.65) adenocarcinomas (results not shown). Restriction to participants without an upper endoscopy at least one year prior to index strengthened the association with gastric cardia cancer (OR 1.87; 95% CI: 1.20, 2.91), but did not materially change the other associations (results not shown).

## Discussion

In this study of esophageal and gastric cancer cases and controls in a community-based population, we found little evidence for an association between oral bisphosphonate use and esophageal squamous cell carcinoma, esophageal adenocarcinoma, or non-cardia gastric adenocarcinoma. There was an adverse association between oral bisphosphonate use and gastric cardia and other gastric adenocarcinomas, however, in the dose-response analysis considering length of exposure to oral bisphosphonates, even just a short exposure to bisphosphonates (less than 12 months) appeared to increase the risk of gastric cardia and other adenocarcinomas, raising the question of biologic plausibility given that it is unlikely that only one year of exposure would increase the risk of cancer. It is possible that the observed association between gastric cardia and other gastric adenocarcinoma may be related to prescribing behaviors of physicians. Within the controls, participants with a history of GERD or a prescription for PPIs or H2-receptor antagonists were more likely to have received an oral bisphosphonate (results not shown), so it is possible that participants at higher risk for gastric cardia or other gastric cancers were more likely to have received a prescription for bisphosphonates leading to spurious associations, although, if this were the case, one would also expect a higher risk of esophageal adenocarcinoma. Bisphosphonates may be associated with gastric ulcers [20], which could potentially be a mechanism for this association, but since there are also indications that bisphosphonates possess antitumor capabilities [21], this finding would need to be replicated.

Previous work has been conflicting relating to the potential association between bisphosphonate use and the risk of esophageal and gastric cancer, but in general, a number of studies support our null findings with esophageal and non-cardia gastric cancer with little support for a positive association with gastric cardia and other gastric cancer risk. A matched cohort study using the UK General Practice Research Database (GPRD) did not detect associations between bisphosphonate use and esophageal cancer, nor an association with esophageal and gastric cancer combined [8]. However, a nested case-control study also using the GPRD found an increased risk of esophageal cancer, but not gastric cancer, with at least one bisphosphonate prescription versus no prescriptions (OR 1.30; 95% CI: 1.02, 1.66) and a higher risk of esophageal cancer for longer durations of use [10]. The reason for the different findings in these two studies is unclear but could reflect the differences in the time period studied, adjustment for confounders, study design, or exclusion criteria. Two additional studies used the GPRD database to address these differing results, again, with contrasting conclusions. In the first study, a statistically significant association with esophageal (OR 1.43; 95% CI: 1.16, 1.75) and all upper gastrointestinal cancers (OR 1.24; 95% CI: 1.06, 1.45) was observed for women and a significant decreased risk of upper gastrointestinal cancer was observed for men (OR 0.75; 95% CI: 0.57, 0.98), but no association was detected with gastric cancer for men and women and with esophageal cancer for men [16]. However, a more recent study using the GPRD and another UK database, QResearch, generally did not find an association between bisphosphonate use and risk of esophageal or gastric cancer [15]. Two studies each were also published from Denmark and Taiwan, with conflicting results. In Denmark, one study found an increased risk of esophageal cancer after treatment with alendronate (hazard ratio (HR) 2.10; 95% CI: 1.01, 4.35) and with etidronate (HR 1.99; 95% CI: 1.24, 3.18), but no dose or time trends were observed [14]. The other Danish study detected a decreased risk of gastric (HR 0.61; 95% CI: 0.39, 0.97) and gastric and esophageal cancer combined (HR 0.63; 95% CI: 0.45, 0.87) for alendronate users, but no association with esophageal cancer only [7]. This study also found that alendronate users were significantly more likely to have undergone a recent upper endoscopy, which could appear to increase the likelihood of diagnosis of esophageal cancer, but the mortality rate would remain the same or be reduced since earlier diagnosis of esophageal cancer would lead to an apparent increase in incidence but cases would be less likely to die due to the earlier diagnosis. In support of this, they found that the short term mortality risk (i.e., within 3 years of initiating alendronate treatment) was decreased for esophageal and gastric cancer [7]. In Taiwan, one study did not detect a significant risk of esophageal cancer with bisphosphonate use [9], while the other study found inverse trends for duration of use among rare and frequent users of bisphosphonates compared to non-users and a non-significant positive trend among regular users [11].

There are limitations to our study. The data on prescription drugs and comorbid conditions were obtained from claims data which may not entirely cover each participant’s healthcare experience. For prescription drugs, including oral bisphosphonates, there is no information on adherence or whether participants took the medication according to instructions. However, since these are filled prescriptions, it is unlikely that participants continued filling the prescription if they were not taking them. Similarly, any bisphosphonate use from another insurance provider would not be included so the total duration of exposure may be underestimated. Comorbid conditions may also be underestimated since they require a physician’s diagnosis in the time period of study. This study only included participants with comprehensive health insurance coverage and therefore, may not be representative of the uninsured or underinsured; however, this population is generally representative of the region of coverage [17]. Finally, residual confounding by unmeasured covariates may be present. For example, we did not have information related to *Helicobacter pylori* seropositivity, which is an important risk factor for gastric cancer. However, although we excluded BMI, a potential confounder, from the primary analyses due to a high proportion of missing data, when we conducted the analyses within the population with valid BMI data, no residual confounding was generally detected. Although we included information on sub-site and histology, we were unable to characterize the tumors using molecular pathological epidemiologic classifications as has been suggested in some recent publications [22–26]. Future studies may wish to consider these molecular pathological classifications, if possible.

This study also has a number of strengths. This data represents a large number of cases and controls from a comprehensive healthcare system. Almost all enrollees of Kaiser Permanente, Northern California receive all health care through this health plan, so it is unlikely that missing utilization or prescription data substantially biased the results. All of the cancer diagnoses were obtained from the Kaiser Permanente, Northern California cancer registry which is a well-established registry with >98% capture of cancer diagnoses compared with validation studies. In addition, we were able to consider histological and site-specific information which few previous studies have considered. Also, since undiagnosed upper gastrointestinal cancer may lead to increased diagnoses of related conditions and new medication uptake and recent initiation of a prescription medication is unlikely to contribute to cancer development, we excluded diagnoses and prescription medications which were first observed in the year prior to the index date.

In conclusion, we did not observe an association between oral bisphosphonate use and esophageal cancer risk within a large community-based population. A significant association was detected with gastric cardia and other gastric adenocarcinoma risk, but this needs to be replicated since no previous studies detected an adverse association with gastric cancer. Future research should include detailed histological and site specific information to further ascertain these possible associations.

## Abbreviations

95% CI: 95% confidence interval
BMI: Body mass index
CCI: Charlson Comorbidity Index
GERD: Gastroesophageal reflux disease
GPRD: General Practice Research Database
HR: Hazard ratio
HRT: Hormone replacement therapy
NSAID: Non-steroidal anti-inflammatory drug
OR: Odds ratio
PPI: Proton pump inhibitor
